# Volumetric modulated arc radiotherapy sparing the thyroid gland for early-stage glottic cancer: A dosimetrical analysis

**DOI:** 10.3892/ol.2014.2039

**Published:** 2014-04-04

**Authors:** EUN SEOK KIM, SEUNG-GU YEO

**Affiliations:** Department of Radiation Oncology, Soonchunhyang University College of Medicine, Cheonan, Chungnam 330-721, Republic of Korea

**Keywords:** carotid artery, early stage, glottic cancer, thyroid gland, volumetric modulated arc therapy

## Abstract

Previous studies on advanced radiotherapy (RT) techniques for early stage glottic cancer have focused on sparing the carotid artery. However, the aim of the present study was to evaluate the dosimetric advantages of volumetric modulated arc therapy (VMAT) in terms of sparing the thyroid gland in early-stage glottic cancer patients. In total, 15 cT1N0M0 glottic cancer patients treated with definitive RT using VMAT were selected, and for dosimetric comparison, a conventional RT plan comprising opposed-lateral wedged fields was generated for each patient. The carotid artery, thyroid gland and spinal cord were considered organs at risk. The prescription dose was 63 Gy at 2.25 Gy per fraction. For the thyroid gland and carotid artery, all compared parameters were significantly lower with VMAT compared with conventional RT. For the thyroid gland, the median reduction rates of the mean dose (D_mean_), the volume receiving ≥30% of the prescription dose (V_30_) and the V_50_ were 32.6, 40.9 and 46.0%, respectively. The D_mean_ was 14.7±2.6 Gy when using VMAT compared with 22.2±3.9 Gy when using conventional RT. The differences between the techniques in terms of planning target volume coverage and dose homogeneity were not significant. When considering a recent normal tissue complication probability model, which indicated the mean thyroid gland dose as the most significant predictor of radiation-induced hypothyroidism, the dosimetric advantage shown in this study may be valuable in reducing hypothyroidism following RT for early stage glottic cancer patients.

## Introduction

For early-stage (T1-2N0M0) glottic cancer, radiotherapy (RT) and surgery have been demonstrated to yield significant local disease control; >90% for T1 and 80% for T2. RT is usually the preferred treatment modality, as it has shown a trend towards improved post-treatment functional outcomes, including voice quality, however, a prospective randomized trial has not been performed (1).

Such favorable outcomes of RT have been achieved using simple RT techniques of parallel-opposed lateral beams with wedges or tissue compensators (2). However, advanced RT techniques, including intensity-modulated RT (IMRT) and volumetric modulated arc therapy (VMAT), have been adopted rapidly for various head and neck cancers. By contrast, RT techniques used for early-stage glottic cancer have not changed considerably, possibly since the target volume is relatively small and involves no elective nodal irradiation, with low rates of severe toxicity (2,3). One previous study questioned the role of IMRT in early-stage glottic cancer (4).

The carotid artery, which is located adjacent to the larynx, is often overlooked, but has recently received attention in several studies that have analyzed the use of advanced RT techniques for patients with early-stage glottic cancer (5–10). RT has emerged as a significant risk factor for carotid artery stenosis and ischemic stroke in head and neck cancer patients (11,12). The thyroid gland is also located close to the larynx, and hypothyroidism is a late complication frequently observed following RT to the head and neck region (13–15). The lateral beams of conventional RT pass through a cranial portion of the thyroid gland, however, to date, no study has investigated the benefits of advanced RT techniques regarding this organ in early-stage glottic cancer patients.

The aim of the present study was to investigate the dosimetric advantages of VMAT compared with conventional RT, in terms of sparing the thyroid gland in patients with early stage glottic cancer.

## Materials and methods

### Patients

A total of 15 patients with cT1N0M0 squamous cell carcinoma of the larynx treated with definitive RT using VMAT between 2011 and 2012 were selected. The staging workup included direct laryngoscopy, computed tomography (CT) and ^18^F-fluorodeoxyglucose positron emission tomography. The clinical stage was determined according to the American Joint Committee on Cancer staging system, 7^th^ edition (16). All patients were male, with a median age of 66 years (range, 55–76 years).

The patients were immobilized in the supine position with a thermoplastic head and neck mask that included the shoulders to ensure reproducibility of treatments. The planning CT scans were performed using a 16-slice CT scanner (Brilliance CT Big Bore; Philips Medical Systems, Cleveland, OH, USA) with a 0.2-cm slice thickness. Intravenous contrast was used in all patients and all RT plans were generated using the Eclipse treatment planning system (Varian Medical Systems, Palo Alto, CA, USA). Treatment was conducted using a Novalis^®^ Tx system (Varian Medical Systems, Palo Alto, CA, USA and BrainLab, Feldkirchen, Germany) and patient set-up was verified prior to treatment; daily by ExacTrac^®^ (BrainLab) and weekly by cone-beam CT. Patients provided written informed consent.

### RT planning

The clinical target volume included the false and true vocal cords, the anterior and posterior commissure, the arytenoids and the subglottic region, extending from the superior thyroid notch to the bottom of the cricoid cartilage. No cervical lymph nodes were included electively. The planning target volume (PTV) was produced by the addition of a 0.5-cm isotropic set-up margin surrounding the clinical target volume. The PTV was truncated within 0.5 cm of the skin surface in the patients without anterior commissure involvement. The surrounding organs at risk (OARs), including the carotid artery, whole thyroid gland and spinal cord, were delineated. The right and left common, internal and external carotid arteries were contoured starting from the sternoclavicular joints and extending upward to the base of the skull.

For the purpose of comparison, a conventional RT plan was retrospectively generated for each patient, using two opposed-lateral wedged fields. The wedge angle was selected to achieve the most homogeneous dose distribution in the PTV, and the block edge margin was uniformly 0.5 cm around the PTV. The collimator of the Novalis^®^ Tx system was angled such that the posterior jaw of the lateral fields was parallel to the cervical spine, and two fields were optimally weighted to provide adequate PTV coverage.

For VMAT, the double-arc plan, which has been previously recommended for locally advanced head and neck cancers due to its higher PTV homogeneity, was selected (17). The arc length or gantry span of each arc was adjusted to avoid the OARs where possible. The collimator was rotated to 45° or 315° to minimize the contribution of the tongue-and-groove effect during treatment. Optimizations were performed by interactively adapting the objectives and their priorities to ensure lower doses to the OARs and to improve PTV coverage and homogeneity. Following optimization, the dose calculation was performed using the anisotropic analytical algorithm and Eclipse dose volume optimizer (version 8.9.17; Varian Medical Systems, Palo Alto, CA, USA). All conventional and VMAT plans were generated using 6-MV photons and a high-definition multileaf collimator consisting of 120 leaves, which included 64 2.5-mm central and 56 5-mm peripheral leaves. The prescription dose was 63 Gy at 2.25 Gy per fraction and all plans were normalized so that ≥95% of the PTV received 100% of the prescription dose.

### Statistical analysis

Dose-volume histograms (DVHs) were created for all treatment plans, and specific dose-volume parameters were compared for the PTV, thyroid gland, carotid artery and spinal cord. For the PTV, the minimum dose (D_min_), maximum dose (D_max_), mean dose (D_mean_) and volume receiving ≥105% of the prescription dose (V_105_) were compared to evaluate PTV coverage and dose homogeneity. For the OARs, the following parameters were compared: D_mean_, V_30_ and V_50_ for the thyroid gland; D_mean_, V_35_ and V_50_ for the carotid artery; and D_max_ for the spinal cord. Comparisons between the dosimetric parameters were performed using the non-parametric Wilcoxon signed-rank test. All statistical tests were two-sided and performed using SPSS software (version 14.0; SPSS, Inc., Chicago, IL, USA). P<0.05 was considered to indicate a statistically significant difference.

## Results

**Figure 1:**
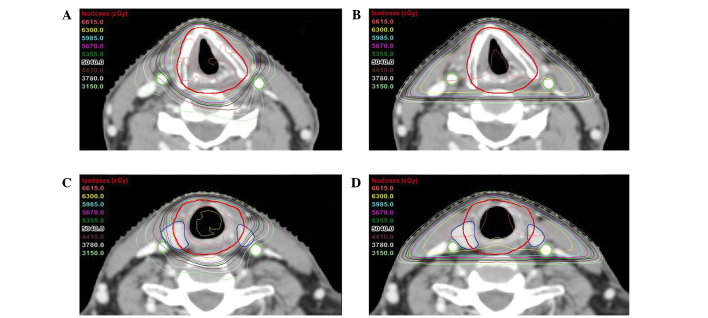
Comparison of the isodose distributions of the two RT techniques in a representative case. (A) VMAT and (B) conventional RT axial plan CT images at the mid-PTV (red) level showing the carotid artery (green). (C) VMAT and (D) conventional RT axial plan CT images at the lower PTV level showing the thyroid gland (blue). RT, radiotherapy; VMAT, volumetric modulated arc therapy; CT, computed tomography; PTV, planning target volume.

**Figure 2:**
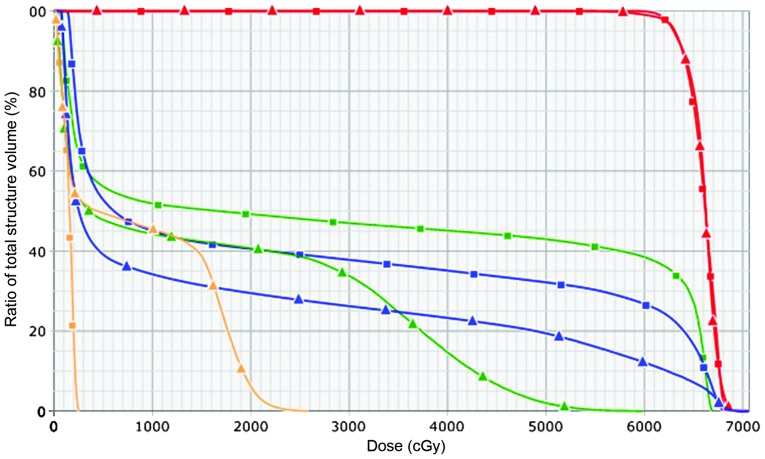
Comparison of dose-volume histograms of the two RT techniques in a representative case [VMAT (triangles) vs. conventional RT (squares)]. The planning target volume (red), carotid artery (green), thyroid gland (blue) and spinal cord (orange) are shown. VMAT, volumetric modulated arc therapy; RT, radiotherapy.

Figs. 1 and 2 show the isodose distributions and DVHs, respectively, according to the RT plan, and Table I shows the dose-volume data for the PTV. For the PTV, no differences were identified in the D_min_ or D_mean_ between VMAT and conventional RT. The median D_min_ and D_mean_ values were 51.0 Gy (80.9% of the prescription dose) and 62.7 Gy (99.5% of the prescription dose) in VMAT, respectively, and 52.3 Gy (83.0%) and 62.8 Gy (99.7%) in conventional RT, respectively. The D_max_ of VMAT tended to be higher when compared with the D_max_ of the conventional RT (P=0.074), and the median D_max_ values were 66.6 Gy (105.7%) and 65.7 Gy (104.3%) for VMAT and conventional RT, respectively. However, V_105_ was not significantly different between the two plans.

Table II shows the dose-volume data for the OARs. For the thyroid gland and carotid artery, all compared parameters were significantly lower in VMAT than in conventional RT. In the thyroid gland, the median reduction rates of the D_mean,_ V_30_ and V_50_ using VMAT were 32.6% (range, 26.5–46.0%), 40.9% (range, 30.7–45.5%) and 46.0% (range, 38.4–67.2%), respectively. In the carotid artery, the median reduction rates of the D_mean_, V_35_ and V_50_ using VMAT were 45.9% (range, 37.0–50.4%), 49.0% (range, 45.0–52.5%) and 92.5% (range, 89.1–100%), respectively.

The measured volume of the entire thyroid gland ranged between 7.4 and 21.9 cm^3^ (median, 14.9 cm^3^). A portion of thyroid gland volume, which was encompassed by lateral beams of conventional RT, ranged between 2.5–7.6 cm^3^ (median, 4.4 cm^3^); on average, it was 32.8% (range, 25.2–40.9%) of the entire thyroid gland volume.

## Discussion

The partial volume effects between the thyroid gland and hypothyroidism were unclear in the study by Emami *et al* (18), which was based on a simple consensus of clinical experience or opinions, with little high-quality clinical data. In addition, the Quantitative Analysis of Normal Tissue Effects in the Clinic reports (19) did not include data concerning the thyroid gland. Recently, a normal tissue complication probability (NTCP) model of radiation-induced hypothyroidism has been developed based on a prospective multivariate analysis (20). The probability of hypothyroidism increases with a higher D_mean_ of the thyroid gland (odds ratio, 1.064/Gy) and decreases with a higher thyroid gland volume (odds ratio, 0.826/cm^3^), indicating that the D_mean_ must be minimized (20). In the present study, VMAT exhibited a median 32.6% reduction in the D_mean_ of the thyroid gland compared with conventional RT. In addition, the absolute reduction of the D_mean_ was a median of 8.0 Gy (range, 4.6–11.1 Gy). Therefore, the VMAT used for early-stage glottic cancer patients may reduce the post-RT risk of developing hypothyroidism, when applying this NTCP model.

Hypothyroidism is a late toxicity that frequently occurs following curative RT to the head and neck region. The incidence of subclinical (high serum thyrotropin) and clinical (high serum thyrotropin and thyroxine) hypothyroidism following RT to the head and neck region varies between 23 and 53% and 11 and 33%, respectively (15). These values are much higher than those observed in a normal population, in which the prevalence of subclinical hypothyroidism is ~8% in females and ~3% in males, and where the prevalence of clinical hypothyroidism varies between 1% and 2% (21). Observation of the thyroid gland using ultrasonography during RT has identified vessel changes, which have been indicated to be associated with late radiation effects on this organ (22). Hypothyroidism is known to develop following a median interval of 1.4–1.8 years, causing a progressively deteriorating quality of life with various clinical symptoms, including, fatigue, weakness, cold intolerance, weight gain, constipation and depression (13,14).

In a study involving only early-stage glottic cancer patients, the post-RT rates of subclinical and clinical hypothyroidism were reported to be 24 and 6%, respectively (23). These rates do not appear to be low considering their relatively small target volume involving no neck node irradiation. Conventional RT consists of opposed-lateral beams to primarily avoid the spinal cord, however, it exposes a certain proportion of the thyroid gland (32.8% on average in the present study) to high radiation doses almost identical to that of the target. To the best of our knowledge, the present study is the first to contour and restrict the dose not only to the carotid artery, but also to the thyroid gland, and revealed the dosimetric benefits of the advanced RT technique in terms of sparing the two organs. A small increase in the D_max_ of the PTV with VMAT may affect larynx function (7), however, the V_105_ was not significantly different between the two techniques. The spinal cord dose was increased, but remained within the recommended tolerable range.

Through acceleration of the atherosclerotic process of the carotid artery, RT may increase the risk of ischemic stroke in head and neck cancer patients (24). The actuarial incidences of cerebrovascular events at 10 years after definitive RT and surgery have been reported as 34% and 26%, respectively (P<0.001) (12). When only the early-stage glottic cancer patients are assessed, RT is also associated with an increased risk of fatal cerebrovascular events compared with surgery, with a cumulative incidence at 15 years of 2.8 versus 1.5% (P=0.024) (25). The results of the present study indicated that VMAT significantly decreases the dose to the carotid artery, which is consistent with previous dosimetric studies of IMRT or VMAT in early-stage glottic cancer (5–10). A total of 35 Gy has been indicated as the threshold for intima media thickness and wall abnormalities of the carotid artery (26). In the present study, the V_35_ was reduced by almost half (49%) with VMAT. An additional radiation-related carotid toxicity is carotid blowout syndrome, a rare but devastating complication following reirradiation of the head and neck region for recurrence or a second malignancy (27). Therefore, it is important to minimize the unnecessary dose to the carotid artery during initial treatment.

In conclusion, compared with conventional RT, VMAT yields significantly improved dose-volume parameters of the thyroid gland and carotid artery, with no differences in target coverage or homogeneity. Considering the NTCP model for radiation-induced hypothyroidism (20), not only the carotid artery, but also the thyroid gland must be contoured and protected as an avoidance structure during advanced RT planning for early-stage glottic cancer. Considering the high rates of tumor control and the long-term survival of patients, favorable dosimetric features of organs associated with late-manifesting toxicities may be valuable.

## Figures and Tables

**Table I:** Comparison of the PTV dosimetric parameters between VMAT and conventional RT (n=15).

Parameters	VMAT, mean ± SD (range)	Conventional RT, mean ± SD (range)	P-value^a^
D_min_, Gy	50.0±2.6 (45.1–52.9)	51.6±4.2 (45.2–56.8)	0.214
D_max_, Gy	66.8±1.3 (65.4–70.1)	65.7±0.6 (64.9–66.5)	0.074
D_mean_, Gy	62.8±0.6 (62.1–64.5)	62.8±0.4 (62.0–63.3)	0.236
V_105_, %	1.7±5.4 (0.0–17.0)	0.005±0.01 (0.0–0.05)	0.116

a Wilcoxon signed-rank test.

PTV, planning target volume; VMAT, volumetric modulated arc therapy; RT, radiotherapy; SD, standard deviation; D_min_, minimum dose; D_max_, maximum dose; D_mean_, mean dose; V_105_, volume receiving ≥105% of the prescription dose.

**Table II:** Comparison of the OAR dosimetric parameters between VMAT and conventional RT (n=15).

Parameters	VMAT, mean ± SD (range)	Conventional RT, mean ± SD (range)	P-value^a^
Thyroid gland			
D_mean_, Gy	14.7±2.6 (10.2–18.0)	22.2±3.9 (15.8–26.5)	<0.01
V_30_, %	19.2±4.0 (12.0–26.2)	31.7±6.2 (21.5–37.8)	<0.01
V_50_, %	13.8±3.8 (8.0–19.4)	26.7±5.9 (17.0–32.6)	<0.01
Carotid artery			
D_mean_, Gy	15.7±3.1 (10.6–20.2)	28.8±6.9 (16.8–39.9)	<0.01
V_35_, %	21.1±6.4 (11.6–32.3)	41.1±10.5 (24.1–58.7)	<0.01
V_50_, %	2.8±2.0 (0.0–6.0)	38.2±10.2 (22.0–55.2)	<0.01
Spinal cord			
D_max_, Gy	29.8±1.9 (25.9–33.0)	3.3±0.8 (2.1–4.8)	<0.01

a Wilcoxon signed-rank test.

OAR, organ at risk; VMAT, volumetric modulated arc therapy; RT, radiotherapy; SD, standard deviation; V_30_, volume receiving ≥30% of the prescription dose; D_mean_, mean dose; D_max_, maximum dose.
